# Prospect of Alpha-Synuclein (A-Syn) Isolation From Saliva as a Promising Diagnostic Biomarker Alternative in Parkinson’s Disease (PD): A Systematic Review

**DOI:** 10.7759/cureus.29880

**Published:** 2022-10-03

**Authors:** Pratyusha Muddaloor, Michelle Farinango, Akhil Ansary, Amulya Dakka, Zahra Nazir, Humaira Shamim, Marie Jean, Muaaz Umair, Safeera Khan

**Affiliations:** 1 Internal Medicine, California Institute of Behavioral Neurosciences & Psychology, Fairfield, USA; 2 Research, California Institute of Behavioral Neurosciences & Psychology, Fairfield, USA; 3 Internal Medicine Clinical Research, California Institute of Behavioral Neurosciences & Psychology, Fairfield, USA; 4 Dermatology, California Institute of Behavioral Neurosciences & Psychology, Fairfield, USA; 5 Psychiatry, California Institute of Behavioral Neurosciences & Psychology, Fairfield, USA

**Keywords:** neurodegenerative disorder, salivary alpha-synuclein, saliva, alpha-synuclein oligomer, parkinson' s disease

## Abstract

Neurodegenerative diseases, in particular Parkinson’s disease (PD), a disabling disorder, require early attention due to the course the diseases take. By the time of clinical manifestation, dopaminergic neuron death would have already exceeded a damaging level. Therefore, the discovery of biomarkers that will effectively diagnose PD at an early stage and help monitor disease advancement is crucial. Out of the available biomarkers and bodily sources from which these can be isolated; alpha-synuclein (a-syn) from saliva seems to be a promising and easily accessible option. This has been further investigated in this systematic review.

A comprehensive literature search on PubMed, PubMed Central (PMC), and Science Direct resulted in 1,439 articles. After screening and exclusion, 12 relevant articles were derived.

In many of the studies, there was a decrease in total salivary a-syn in PD patients compared to healthy controls (HC), with an increase in oligo a-syn and oligo a-syn/total a-syn ratio as a rather consistent finding amongst the studies reviewed. On the other hand, a few studies revealed no significant difference in a-syn levels between the controls and PD patients. Another common finding was the lack of disease severity correlation with the marker, probably due to the scarcity of longitudinal studies conducted and smaller cohorts recruited in the studies.

Overall, the total a-syn did show a genetic and phenotypic association, whilst oligo a-syn had the potential to serve as a biomarker for disease diagnosis. With the standardization of sample collection methods and diagnostic tools, and the accomplishment of longitudinal studies, further importance of salivary a-syn as a biomarker in PD could be established, utilizing the already existing data as an encouraging foundation for future research.

## Introduction and background

Parkinson's disease (PD), the second most common neurodegenerative movement disorder, affects 1-2 per 1,000 people at any time, that is, more than six million people worldwide, and it is estimated that this number will double by the year 2040, making PD the fastest-growing neurodegenerative disease [1-2]. With its prevalence increasing, it is a topic with ample scope for investigating methods of early detection and screening. 

PD is the clinical outcome of progressive degeneration of central and peripheral nervous system cells, which ultimately leads to cognitive and motor function deficits. Degeneration of dopaminergic neurons with subsequent decrease in dopamine in the substantia nigra, along with intracytoplasmic accumulation of alpha-synuclein (a-syn) protein aggregates called Lewy Bodies (LB), are major hallmarks of the disease [3]. It is these aggregates that form the pathological basis of the disease. The diagnosis of PD is based on the clinical findings of bradykinesia in addition to resting tremors and/or rigidity. Unfortunately, by this stage, up to 50% of dopaminergic cell bodies and close to 80% of striatum dopaminergic terminals are already irreversibly lost, even if physical manifestations fail to parallel with histopathological findings [4]. A lesser known fact is that parts of the body such as salivary glands, gastroenteric plexuses, adrenal glands, and the urinary system seem to be involved in PD pathology long before motor symptoms develop [5].

A-syn, being a major component of LB, can be found in peripheral tissues and body fluids, and is readily secreted into extracellular spaces, partly being released into exosomes, making it a promising biomarker to detect PD earlier and monitor disease progression [6]. In physiological conditions, a-syn is prevalently expressed as a monomeric form (a-synmon), localized in the cytoplasm and the cellular nuclei or bound to the synaptic vesicles. In PD, a-synmon aggregates into a-syn oligomers (oligo a-syn) followed by conversion into mature amyloid fibrils, eventually forming LB [7]. The two most commonly assessed physiological fluids that are used for the detection of markers of PD are cerebrospinal fluid and blood. Major drawbacks to these methods include patient feasibility for repeated testing owing to invasiveness and the possibility of blood contamination. Subsequently, more readily accessible bio- fluids, such as urine or saliva, are attractive alternatives [6]. 

Saliva to test a-syn is a suitable biomaterial that can be used as a diagnostic method because it is relatively easy to obtain, its non-invasive nature, simple processing, cost-effectiveness and lower protein content than blood or urine [3]. Additionally, salivary flow and composition are regulated by the autonomic nervous system, suggesting a direct relationship between saliva and the nervous system, allowing specific biomarkers linked to neurodegenerative diseases to be detected in saliva [3]. Salivary a-syn holds promise as a novel potential biomarker for PD, although, studies on native a-syn content in human saliva and its role in clinical features of PD have yielded conflicting results, further in-depth investigations in the future to establish a relationship will be required [8].

In this systematic review, we aim to explore the role and reliability of salivary a-syn as a diagnostic biomarker in patients with PD.

## Review

Methods

This systematic review was designed and conducted adhering to the Preferred Items for Systematic Reviews and Meta-Analysis (PRISMA) 2020 guidelines [9].

Search strategy

We searched PubMed/MEDLINE, PubMed Central (PMC), and Science Direct thoroughly to identify full-text relevant published papers. These databases were rigorously searched using appropriate keywords with relevant BOOLEANS and Medical Subject Headings (MeSH) terms to find all potentially relevant articles illustrating the role of salivary a-syn as a diagnostic biomarker in patients with PD. The MeSH strategy used in PubMed and PMC was: (("saliva"[MeSH Terms] OR "saliva"[All Fields]) AND ("alpha-synuclein"[MeSH Terms] OR "alpha-synuclein"[All Fields] OR ("alpha"[All Fields] AND "synuclein"[All Fields]) OR "alpha synuclein"[All Fields])) AND ("parkinson disease"[MeSH Terms] OR ("parkinson"[All Fields] AND "disease"[All Fields]) OR "parkinson disease"[All Fields] OR ("parkinson's"[All Fields] AND "disease"[All Fields]) OR "Parkinson’s disease"[All Fields]). For Science Direct, keywords "salivary alpha synuclein AND parkinsons" were used to gather data.

After gathering all papers, references were meticulously searched to ensure that no potentially relevant publications were left unnoticed. The titles, abstracts and subject headings were reviewed for relevance.

Inclusion and exclusion criteria

We included articles published in English relevant to the pertaining topic from the January 2016 to December 2022. All types of papers including randomized and non-randomized clinical trials, observational studies, and traditional and systematic reviews were included. The primary focus was on the adult and geriatric population diagnosed with PD. Articles focused on the pediatric population, unpublished or grey literature, and animal studies were excluded.

Data extraction

Data selection and extraction were carried out individually by two researchers (first and second authors). Any disagreements were resolved by reviewing the study design, checking relevance to inclusion and exclusion criteria, and the outcome of the study. In circumstances where a unanimous decision could not be made, we solicited the assistance of a third reviewer.

Quality assessment of the studies

Traditional reviews were critically appraised with the help of the scale for the quality assessment of narrative review articles (SANRA) checklist [10], while the systematic reviews were evaluated by the Assessment of Mutliple Systematic Reviews (AMSTAR) tool [11]. Additionally, the Joanna Briggs Institute (JBI) critical appraisal checklist was used for case reports and observational studies [12]. 

In the SANRA checklist, for each of the six criteria, a score out of two was reported. A score of ten or above was considered a high-quality study and included in the systematic review. Regarding the AMSTAR and JBI checklists, each criterion was given a yes/partial yes/no. A maximum of 30% bias was tolerated, excluding all other studies that fell short of this. The quality assessment tables containing each article assessment are shown in Tables 1, 2, and 3 below.

**Table 1:** SANRA checklist for traditional reviews SANRA: Scale for the quality assessment of narrative review articles Criteria sourced from Baethge et al [10].

SANRA checklist	Traditional reviews				
	Polissidis et al. [2]	Pawlik et al. [3]	Leggio et al. [4]	Farah et al. [13]	Schepici et al. [14]
Important to reader	2	2	2	2	2
Aims of narrative reviews	2	2	2	2	2
Comprehensive literature search description	2	2	0	0	1
Referencing	2	2	2	2	2
Scientific reasoning	2	2	2	2	2
Appropriate presentation of data	2	2	2	2	2
Total	12/12	12/12	10/12	10/12	11/12

**Table 2:** AMSTAR checklist for systematic reviews AMSTAR: Assessment of multiple systematic reviews; RoB: Risk of bias; PICO: Population, intervention, control, outcome Criteria sourced from Shea et al [11].

AMSTAR checklist	Systematic review
	Figura et al. [5]
PICO included in the research question and inclusion criteria?	yes
Explicit statement to show review methods were established prior to conduct of the review? Any justification of significant deviations from the protocol?	no
Did the review authors explain their selection of the study designs for inclusion in the review?	no
Utilization of a comprehensive literature search strategy?	partial yes
Was study selection performed in duplicate by review authors?	yes
Was data extraction performed in duplicate by review authors?	yes
Was a list of excluded studies with justifications provided by the review authors?	yes
Were the included studies described in adequate detail?	partial yes
Was a satisfactory technique used for RoB assessment in individual studies include in the review?	no
Did the review authors report on the funding sources for the included studies in the review?	no
Usage of appropriate methods for statistical combination of results if a meta-analysis was performed?	no meta-analysis conducted
If meta-analysis was performed, was the potential impact of RoB assessed with regard to individual studies?	no meta-analysis conducted
Did the review authors account for RoB in individual studies when interpreting/discussing the results of the review?	no
A satisfactory explanation for any heterogeneity observed in the results of the review given?	yes
If they performed quantitative synthesis did the review authors carry out an adequate investigation of publication bias and discuss its impact on the results of the review?	no meta-analysis conducted
Were any potential sources of conflict of interest, including funding they received for conducting the review reported?	no

**Table 3:** JBI checklist for observational cross-sectional studies JBI: Joanna Briggs Institute checklist Criteria sourced from Moola et al [12].

JBI checklist for observational cross-sectional studies	Vivacqua et al. [7]	Goldmann et al. [6]	Chahine et al. [15]	Fernández-Espejo et al. [8]	Cao et al. [16]	Vivacqua et al. [17]	Kang et al. [18]
Were the criteria for inclusion in the sample clearly defined?	yes	yes	yes	yes	yes	yes	yes
Were the study subjects and the setting described in detail?	yes	yes	yes	yes	yes	yes	yes
Was the exposure measured in a valid and reliable way?	yes	yes	yes	yes	yes	yes	yes
Were objective, standard criteria used for measurement of the condition?	yes	yes	yes	yes	yes	yes	yes
Were confounding factors identified?	yes	yes	yes	yes	yes	yes	yes
Were strategies to deal with confounding factors stated?	yes	yes	unclear	no	unclear	unclear	yes
Were the outcomes measured in a valid and reliable way?	partially yes	yes	unclear	yes	yes	yes	yes
Was appropriate statistical analysis used?	yes	yes	yes	yes	yes	yes	yes

Results

**Figure 1:**
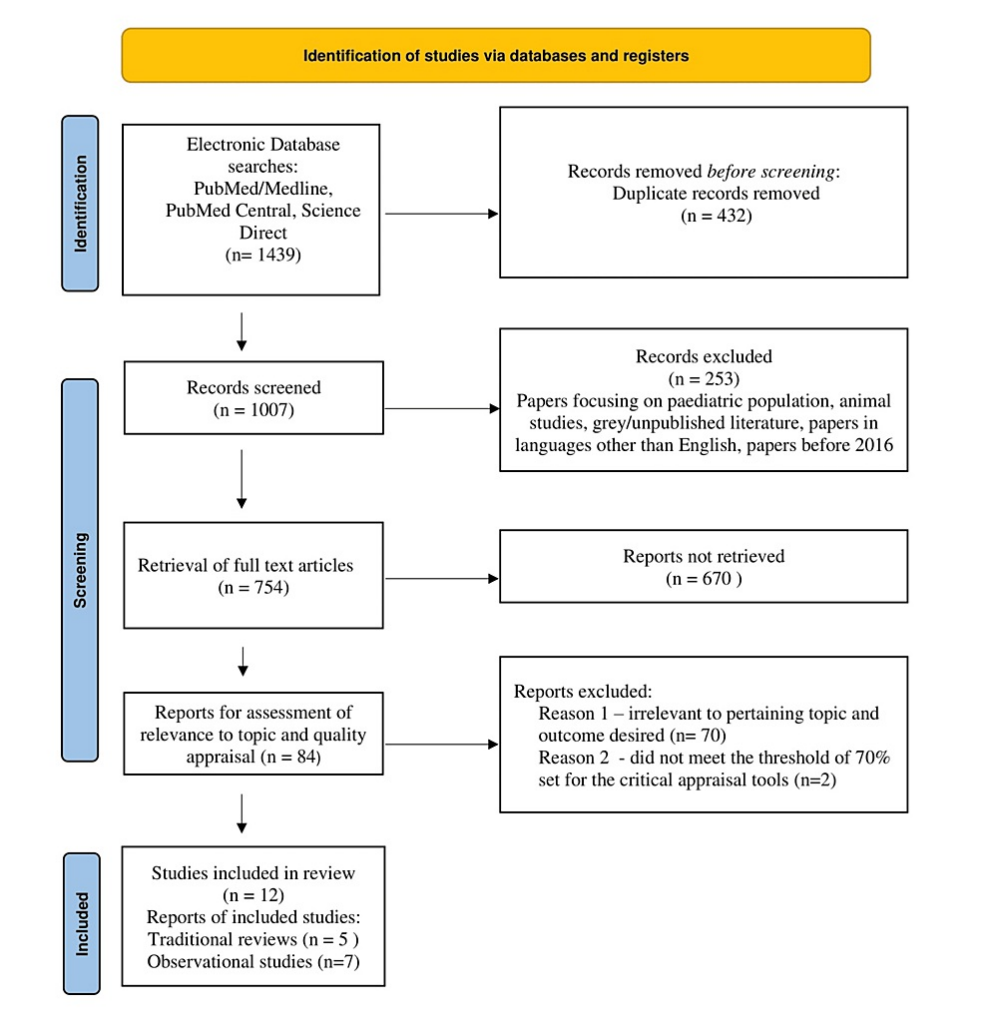
PRISMA 2020 flow diagram for systematic reviews, which included searches of databases only PRISMA: Preferred items for systematic reviews and meta-analysis Criteria sourced from Page et al [9].

A total of 1,439 articles were found using multiple search strategies. Out of these 1,439 articles, 37 were from PubMed, 992 from PMC, and the remaining 410 from Science Direct. After the removal of 432 duplicates and screening according to the inclusion and exclusion criteria, 685 papers were dropped. Consequently, 754 papers were left, and after checking for availability of full texts, and filtering through titles and abstracts, we were left with 84 papers. Of these articles, 12 articles were finalized using critical appraisal tools and based on the intervention and outcome to be studied in our systematic review, we were left with five traditional reviews, and seven observational studies. A complete PRISMA flow diagram is shown below in Figure 1 [9].

Discussion

Neurodegenerative disorders such as PD depict a devastating progression if not detected and controlled at an early stage. Therefore, it is imperative to find means of diagnosing this disease with high specificity and discover an objective measure for disease progression. Owing to patient discomfort with the usage of cerebrospinal fluid and frequent blood draws for biomarker testing, novel markers such as saliva have been investigated. The superiority of using saliva as a marker over the other two forms can be explained by the ease of access and non-invasive, painless nature, not to mention the low cost and lower stress faced by the patient during sample collection, as no hospitalization is required [3,13]. 

An already known fact being a hallmark of PD is the increased concentration of a-syn aggregates in LB, a manifestation of a-syn gene (SNCA) multiplication [4,13]. Additionally, point mutations in the SNCA gene sequence have shown to affect a-syn aggregation resulting in two forms: an oligomeric aggregate, associated with numerous organelle dysfunction and impairment in the axonal transport system, and the fibrillar insoluble aggregates forming LB [4,13]. Another effect of a-syn responsible for the spread of disease is the effect on neighbouring cells stimulating the aggregation of endogenous a-syn [4]. A proportion of a-syn is transported within extracellular vesicles (EV), paralleling the pathology of the host cell, and is resistant to degradation owing to the external lipid bilayer and impaired proteolytic activity of lysosomes in PD patients. Emphasis on a-syn-EVs is because brain-derived EVs can transport their material across the blood-brain barrier (BBB) into the systemic circulation, in turn, available for isolation. Another point to mention is the function of EVs wherein they mediate the spread of a-syn between cells and, once within target cells, relay the a-syn to lysosomes for final degradation [4].

The role of salivary a-syn as a biomarker in patients with PD can be discussed by comparing the concentrations of a-syn total and oligo a-syn that have been found throughout several studies. A recent review by Pawlik et al. in 2021 showed that although there was a decrease in the total a-syn in PD, no correlation existed between a-syn total and disease severity. However, an inverse relation with the age of PD patients was noted, not to mention an association of total a-syn with specific genes. The review also revealed an increase in both oligo a-syn and oligo a-syn/a-syn total ratio in PD patients versus healty controls (HC). A conflicting finding in some studies showed an insignificant difference in the total a-syn levels between PD and HC [3]. A similar finding was seen in the review by Shepici et al. in 2020 [6], backed up by Chahine et al. and Goldman et al. [14,15]. 

In contrast to the previous findings, in 2021, Leggio et al, in a study of EVs displayed an increase in salivary a-syn-EVs in PD patients. The other EV findings comprised an increase in oligo a-syn and oligo a-syn/a-syn total ratio, as well as no disease severity association with salivary EV-oligo a-syn forms [4]. A similar EV ratio finding was seen by Cao et al. in 2019 [16]. In a 2021 study involving 45 PD participants and 30 HC, levels of a-syn in saliva were found to be similar in both subsets, in addition to a similar saliva/serum a-syn ratio in both groups. However, this study did agree with the fact that no association existed between a-syn and disease progression and clinical features [8].

In 2020, Polissidis et al. and Shepici et al., in their reviews, concurrently noted a decrease in total a-syn in PD, an increase in oligo a-syn, and an increase in oligo a-syn/a-syn total ratio in PD as compared to HC [2,14]. The same was reported in 2019 by Vivacqua et al. in their study of 100 PD patients and 80 HC, with an added finding of no relation between total a-syn and oligo a-syn and severeness of disease, in line with a study comprising 74 PD subjects and 60 HC by Cao et al. in 2019 [16,17]. Quite opposite to this, a positive correlation of a-syn total with disease severity was discovered, with lower values at early stages and higher values seen at later stages [13]. No such correlation was seen with the oligomeric form. In agreement with the previous two results, a cross-sectional study was conducted including by Vivacqua et al. [7]. Other findings from Farah et al. and Vivacqua et al. were in conjunction with the conclusions of Polissidis et al. and Shepici et al. [2,7,13,14].

An observational study that recruited 201 PD patients and 67 HC by Kang et al. in 2016 revealed no significant difference between a-syn total between both groups as well as no disease or gender or pharmacotherapy correlation with the marker. Despite this finding, an inverse relationship existed with gender and some association was found with certain gender subtypes. Furthermore, an increase in oligo a-syn was detected and the relation between oligo a-syn/a-syn total ratio and stage of PD was established, with a low ratio early on and a high ratio in the later stages [18]. 

An explanation for several of the results has been documented by Vivacqua et al. in their 2016 study. The study explains that in normal physiological conditions, a balance exists between the monomer (a-syn total) and the oligomer forms of a-syn. This balance is disrupted in PD leading to increased aggregation and accumulation of oligo a-syn throughout the body. As the monomer forms are consumed during aggregation, a decreased level of a-syn total is reported in saliva [7]. A summary of the findings has been tabulated in Table 4 below.

**Table 4:** Salivary a-syn biomarker associated with PD reported in several studies PD: Parkinson's disease; HC: Healthy controls; ELISA: Enzyme-linked immunosorbent assay; a-syn: Alpha-synuclein; oligo a-syn: Oligomeric alpha-synuclein; EV: Extracellular vesicles

Study	Study participants	Methods of testing for biomarker in saliva	Results
Pawlik et al. 2021 [3]	PD: 418, HC: 232	ELISA, Luminex assay, Western Blot, Magnetic bead-based Luminex assay	↓ total a-syn in PD
			No significant difference in total a-syn between PD and HC
			No correlation in a-syn total with disease severity
			↓ total a-syn with age in PD not HC
			Total a-syn associated with specific g-alleles
			↑ oligo a-syn in PD
			↑ oligo a-syn/a-syn total in PD
Leggio et al. 2021 [4]	PD: 74, HC: 60	Western Blot, Nanosight 300	↑ oligo a-syn in PD salivary-EVs vs HC
			↑ oligo a-syn/a-syn total ratio in PD
			No association of salivary EV oligomeric a-syn with severity of disease
			↑ a-syn in salivary-EVs in PD vs HC
Fernandez-Espejo et al. 2021 [8]	PD: 45, HC: 30	ELISA	Native a-syn in saliva similar in both PD and HC
			No correlation with a-syn and clinical features of PD
			Saliva/serum a-syn ratio similar in PD and HC
Polissidis et al. 2020 [2]	PD: 134, HC: 100	ELISA, Western Blot, Nanosight 300	↓ total a-syn in PD vs HC
			↑ oligo a-syn in PD
			↑ oligo a-syn/a-syn total ratio in PD vs HC
			oligo a-syn not associated with disease severity
Shepici et al 2020 [14]	PD: 303, HC: 438	ELISA, Electrochemiluminescence assay, Western Blot, Immunohistochemical analysis	↓ total a-syn in PD vs HC
			↑ oligomeric a-syn in PD vs HC
			↑ oligo a-syn/a-syn total ratio in PD vs HC
			No significant difference in total a-syn in PD vs HC
Chahine et al. 2020 [15]	PD: 59, HC: 21	ELISA	No significant difference in a-syn between PD and HC
Vivacqua et al. 2019 [17]	PD: 100, HC: 80	ELISA, _olig_ELISA2	↓ total a-syn in PD vs HC
			↑ oligo a-syn in PD vs HC
			↑ oligo a-syn/total a-syn ratio in PD
			No correlation of a-syn total, oligo a-syn with disease severity
Cao et al. 2019 [16]	PD: 74, HC: 60	Western Blot, Nanosight 300	↑ oligo a-syn in PD vs HC
			↑ oligo a-syn/a-syn total in PD salivary EVs than HC
			No significant difference in a-syn total between PD and HC
			No correlation of oligo a-syn, or oligo/total ratio with disease severity
Farah et al. 2018 [13]	PD: 104, HC: 85	Western Blot, Spectrometry, Lumex assay, ELISA	↓ a-syn total in saliva of PD vs HC
			↑ oligo a-syn in PD
			↑ oligo a-syn/a-syn total ratio in PD vs HC
			a-syn total positively correlates with disease progression (lower values: early disease; higher values: late disease)
			No disease correlation with oligo a-syn
Goldman et al. 2017 [6]	PD: 115, HC: 88	ELISA	Salivary a-syn did not differ between PD and HC
Kang et al. 2016 [18]	PD: 201, HC: 67	Luminex assay, Gel filtration chromatography, Western Blot	No difference between total a-syn between PD and HC
			No correlation between total a-syn and disease severity/phenotype/gender/pharmacotherapy
			↓ total a-syn with age in PD vs HC
			Total a-syn displays genetic subtype correlation (to be further investigated)
			↑ oligomeric form of a-syn in PD vs HC
			Low oligo a-syn/a-syn total ratio in early stages of disease
			High oligo a-syn/a-syn total ratio in late stages of PD
Vivacqua et al. 2016 [7]	PD: 60, HC: 40	ELISA	↓ salivary total a-syn in PD vs HC
			↑ oligo a-syn in PD vs HC
			↑ oligo a-syn/total a-syn ratio in PD
			Positive correlation between a-syn total and disease duration and severity
			No correlation between disease severity and oligo a-syn

Limitations

A few important limitations to be noted when considering saliva as a sample to test a-syn as a biomarker include a lack of standardization and specificity of sample collection and biomarker testing as reported by Pawlik et al. [3]. The antibodies used in the enzyme-linked immunosorbent assay (ELISA) diagnostic test are targeted mainly to the unaggregated forms of a-syn, failing to detect the oligomer forms, distorting the a-syn total results obtained in these studies [17]. Another shortcoming is the fact that PD patients face either excessive salivation (hyper sialorrhea) or dry mouth (xerostomia) that influence the collection of saliva as well as its consistency and concentration. Additional to these, the medication used to treat PD, including anticholinergics and tricyclic antidepressants to name a few, also impact salivary flow rate. Local factors like diurnal variation, mouth hygiene and flora, environmental factors like tobacco smoke, ethanol and individual saliva variability have a strong effect on salivary composition too. The physiological presence of proteolytic and glycolytic enzymes in saliva causes proteolytic digestion of a-syn [7]. 

A major drawback in the studies is that they all involve a small cohort group and lack a longer period of study, that is, longitudinal studies to fully study the association of salivary a-syn with disease progression and whether it is of any diagnostic value [3]. Despite trying we could not recover any randomized controlled trials for our review, which would have proven to be supplementary data.

Future Recommendations

Future recommendations comprise the use of larger cohorts studied over a longer period, standardized methods for both sample collection and processing, utilizing early morning non-stimulated saliva post fasting without any recent exposure to tobacco or alcohol, with added protease inhibitors, whilst considering parameters such as salivary flow rate and protein content [3,7]. The development of anti-oligomeric a-syn specific antibodies could strengthen data and future studies could in turn establish the role of a-syn in saliva as a biomarker for early detection and its pattern in PD progression [7].

## Conclusions

Based on several studies that have been compiled, it is evident that salivary a-syn does have a part to play in PD. As PD has a destructive course, wherein clinical diagnosis is made many years after neuronal insult, the discovery of novel markers such as salivary a-syn is imperative to aid in early diagnosis and monitor disease progression for effective management. The emphasis on using saliva to investigate a-syn comes down to the non-invasive nature and easy accessibility for repeated sampling.

In conclusion, majority of the studies disclosed the utilization of oligo a-syn for diagnosis of PD, whilst using a-syn total for classifying PD according to phenotype and genetic makeup. Correlation with disease progression and severity, although showing conflicting results, is an area researchers should investigate in the future to establish a certain relationship. Prospects in this area are promising, given that standardized methods of sample collection and standardized diagnostic tests with high specificity are developed. Future longitudinal studies encompassing a larger group of participants could prove constructive in ascertaining a relation between salivary a-syn levels and disease severity and progression.
